# Sex Differences in the Temporal Recovery of Neuromuscular Function Following Resistance Training in Resistance Trained Men and Women 18 to 35 Years

**DOI:** 10.3389/fphys.2018.01480

**Published:** 2018-10-23

**Authors:** Robert W. Davies, Brian P. Carson, Philip M. Jakeman

**Affiliations:** ^1^Department of Physical Education & Sports Sciences, University of Limerick, Limerick, Ireland; ^2^Food for Health Ireland, Centre for Interventions in Infection, Inflammation & Immunity (4i), University of Limerick, Limerick, Ireland; ^3^Health Research Institute, University of Limerick, Limerick, Ireland

**Keywords:** exercise, fatigue, performance, recovery, sex characteristics, strength

## Abstract

To investigate sex differences in the temporal recovery of neuromuscular function following resistance training (RT), eleven men and eight women 18–35 years completed a single RT bout (barbell back-squats, 80 % 1RM, 5 sets × 5 reps, 25 % duty cycle, then 1 set × max reps). Measures of muscle function (isometric, concentric, eccentric knee extensor strength, and countermovement jump (CMJ) height), serum creatine kinase activity (CK) and lower-body muscle pain were assessed before RT (0 h), +4 h, +24 h, +48 h, and +72 h post-RT. Data are mean % change from PRE (SD) and effect size (ω^2^, d). Men and women had similar RT-experience (men, 2.1 (0.8) years vs. women 2.4 (1.0) years, *P* = 0.746, and *d* = 0.3) and 1RM strength per kg lean mass (men, 1.9 (0.2) kg⋅kg^-1^ vs. women, 1.8 (0.3) kg⋅kg^-1^, *P* = 0.303, and *d* = 0.3). A 36 (12)% increase in lower-body muscle pain was reported following RT (*P* < 0.05, *d* > 0.9). There was an absence of any overt change in CK [+24 h, 74 (41) IU⋅L^-1^; pooled mean (SD)]. Decrements in knee extensor strength and CMJ height were observed +4 to +72 h for both men and women (*P* < 0.05, ω^2^ = 0.19–0.69). Sex differences were apparent for CMJ height (+24 h men, -10 (6)% vs. women, -20 (11)%, *P* < 0.001, and *d* = 1.8) and isokinetic concentric strength (+24 h men, -10 (13)% vs. women -25 (14)%, *P* = 0.006, and *d* = 1.8), with a more pronounced loss and prolonged recovery in women compared to men (e.g., CMJ + 72 h men, -3 (6)% vs. women, -13 (12)%, *P* = 0.051, and *d* = 1.1). We conclude that the different temporal recovery patterns between men and women are not explicable by differences in muscle strength, RT performance, experience, muscle damage or fatigability.

## Introduction

Repeated forceful muscle contractions performed during resistance training (RT) cause a momentary reduction in muscle force, limiting work capacity, and neuromuscular function (Byrne and Eston, 2002). To offset fatigue during RT, periods of work are interspaced with rest. Indeed, factors known to immediately inhibit neuromuscular function (e.g., muscle temperature, pH, electrolyte imbalance, energy availability, and metabolite accrual) are resolved in minutes (Allen et al., 2008), thus enabling a greater volume or intensity of work to be completed during RT (Miranda et al., 2007). However, not all of the fatigue is completely resolved in minutes, as even days following RT cessation an observable reduction in voluntary muscle force persists, affecting normal muscle function (Byrne and Eston, 2002; Izquierdo et al., 2009; McCaulley et al., 2009). The magnitude and temporal recovery from RT evoked fatigue are inextricably linked to the volume, mode, and loading intensity of the RT. Indeed, disruption to the muscle milieu, inflammation, muscle pain, and muscle damage have been observed hours and days following RT cessation, and are associated with neuromuscular dysfunction (MacIntyre et al., 2000; Crameri et al., 2007; Allen et al., 2008; Place et al., 2015).

Men and women have different physiological and neuromuscular characteristics, resulting in marked differences for exercise performance and recovery (Hunter, 2014). Whilst, on average, men are stronger, women are less fatigable, able to sustain force at the same relative intensity for a longer period of time (Hunter et al., 2004, 2006; Wüst et al., 2008; Ansdell et al., 2017). Sex differences in strength and fatigability have been previously attributed to variation in muscle phenotype (Miller et al., 1993). It is reported that women have smaller muscle fibers than men (Miller et al., 1993) and a higher proportion of type I fibers relative to type II (Staron et al., 2000; Roepstorff et al., 2006; Welle et al., 2008), greater muscle capillarisation (Roepstorff et al., 2006) and blood flow during exercise (Parker et al., 2007) with distinct glycolytic and oxidative capacities (Nygaard, 1981; Simoneau and Bouchard, 1989). The difference in strength between equally trained men and women is accounted for by muscle size (Bishop, 1987). And whilst some of the difference in fatigability between sexes is accounted for by strength, muscle phenotype variation is still thought to factor as differences in fatigability are still apparent when men and women are matched for strength (Fulco et al., 1999; Hunter and Enoka, 2001; Hunter et al., 2004, 2006; Wüst et al., 2008; Ansdell et al., 2017). Distinct variation in the muscle phenotype not only affects strength and fatigability but also the regenerative capacity force following contraction (i.e., recovery), having temporal aspects (Allen et al., 2008). We hypothesized that sex may differentially affect the temporal recovery of neuromuscular function following RT, even when men and women were matched for RT-strength and experience.

Understanding the temporal recovery between men and women could have important practical implications, allowing practitioners to make more informed decisions when designing longitudinal training programs. Sex differences for the recovery of neuromuscular function have been previously investigated during and minutes after exercise (Fulco et al., 1999; Hunter and Enoka, 2001; Hunter et al., 2004, 2006; Wüst et al., 2008; Ansdell et al., 2017) and following exercise-induced muscle damage using supramaximal contractile forces (Sayers and Clarkson, 2001; Power et al., 2013). However, the full temporal recovery of neuromuscular function following RT has rarely been examined, and to our knowledge it has not been measured in men and women of similar strength, RT experience and performance. To assess sex differences following RT, we compared the temporal recovery of neuromuscular function in men and women of similar RT-strength and experience. By exercising subjects to a predefined level of volitional exhaustion, we provide direct insight into the impact of sex on the subsequent temporal recovery process.

## Materials and Methods

### Subjects

The study design and all procedures were approved by the University of Limerick Education and Health Sciences Research Ethics Committee (2014_05_11_EHS), conforming to standards set by the Declaration of Helsinki. Subjects were informed of the risks and benefits associated with participation before providing written informed consent. Eligibility criteria was: (i) 18–35 years; (ii) resistance trained (operationally defined as self-reporting at least 0.5 years RT-experience, >3 h⋅wk^-1^ prior to starting the study); (iii) competently perform the back-squat exercise with a minimum level of strength (1.25 kg⋅kg^-1^ body mass one repetition maximum (1RM)); (iv) no current injury or illness; (v) not currently taking any medication or dietary supplements. Sample size estimates of 8–12 were required per group for adequate statistical power (α = 0.05, β = 0.2, and *d* = 1.2) (Byrne and Eston, 2002). Eleven men [23 (3) years, 1.82 (0.06) m, and 83 (3) kg body mass; mean (SD)] and eight women [24 (4) years, 1.71 (0.05) m, and 67 (8) kg body mass; mean (SD)] of similar levels of strength [men, 1.9 (0.2) kg⋅kg lean mass^-1^ vs. women, 1.8 (0.3) kg⋅kg lean mass^-1^; mean (SD)] and RT experience [men, 2.1 (0.8) years vs. women 2.4 (1.0) years] completed the study with no attrition.

### Experimental Design

**FIGURE 1:**
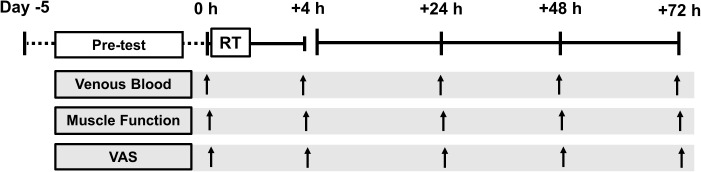
Schematic diagram of the experiment. Pre-test (day –5 to day 0 (0 h)) consisted of: health and exercise screening, one-repetition maximum, 4 days weighed dietary record, dual-energy x-ray absorptiometry and familiarization testing. Tests were conducted at baseline (0600–0900, 0 h) and +4 h, +24 h, +48 h, and +72 h following resistance training (RT).

Subjects reported to the laboratory for testing at the same time each day, fasted overnight (∼ 10 h post-absorptive) between 0600 and 0900 for two consecutive weeks. In the 1st week a preliminary screen (medical and exercise) and measures of muscle function were performed each day to reduce a learning effect from repeat testing. Body composition was measured using whole-body, dual energy x-ray absorptiometry (DXA) (Lunar iDXA, GE Healthcare) and analyzed (enCORE™ v.14.1) according to standard procedure, in line with the international society of clinical densitometry recommendations (International Society of Clinical Densitometry [ISCD], 2015). Following criterion baseline measures (for temporal comparison) at the start of week 2 (0 h) a RT session was performed and subsequent functional measures were obtained +4, +24, +48, and +72 h following RT cessation (Figure 1). For the entire duration of the study subjects were instructed to refrain from any other strenuous physical activity, dietary supplementation, ergogenic, or therapeutic aids (e.g., compression, massage, heat therapy etc.,) which was confirmed via a self-report questionnaire. Subjects were instructed to maintain their habitual dietary intake throughout the study, recording a 4 day weighed food diary in week 1.

### Resistance Training

**FIGURE 2:**
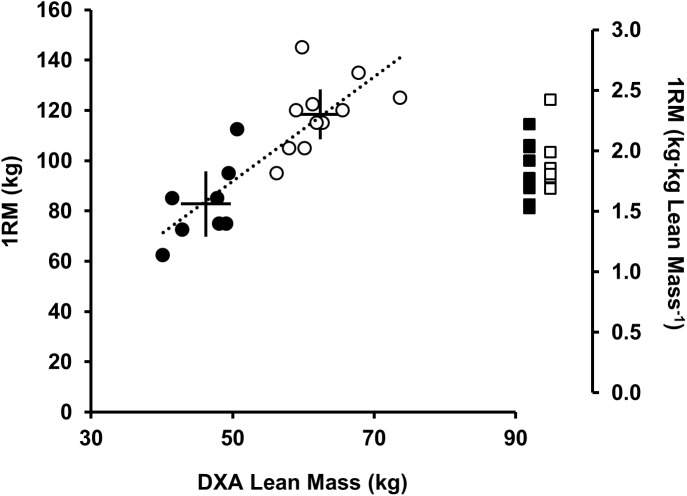
Relationship between DXA lean mass (kg) and 1RM back-squat strength (kg; left axis), men (open circles), and women (black circles). Solid lines represent the mean (intersection) and 90% CI. Dotted regression line shows strong positive correlation between DXA lean mass (kg) and 1RM (kg) (*r*^2^= 0.71, *P* < 0.001). Sex differences were observed for lean mass (kg) and 1RM (kg) (*P* < 0.05) but not 1RM [(kg⋅kg lean mass^-1^) (*P* = 0.401), men (open squares), and women (black squares)].

To increase external and ecological validity we used a barbell back-squat exercise. During the exercise the subject fixes a loaded barbell across the shoulders on the trapezius (above the posterior aspect of the deltoids), flexing the hips and knees until thighs are parallel to the floor, thereafter extending hips and knees to a standing position. Back-squat competency and strength were assessed prior to RT, which were conducted by a certified and experienced strength and conditioning professional. For the RT session each subject completed a 100 w cycle for 5 min then a 1RM barbell back-squat protocol (Newton et al., 2011). The barbell was loaded with 80 % 1RM and subjects back-squatted 5 sets × 5 continuous reps with 90 s rest between sets. A metronome and visible timer was used to time cadence (6 s per rep, 25% duty cycle) and rest. Ninety seven percent of the variance in strength between equally trained men and women is accounted for by the difference in muscle size (Bishop, 1987). Normalizing 1RM strength to muscle size (kg DXA lean mass) provides groups of similar RT-experience, strength (Figure 2) and loading intensity (80% 1RM). To account for differences in fatigability between subjects, one additional set of continuous repetitions was performed to volitional exhaustion (80% 1RM). Volitional exhaustion was operationally defined as the inability to complete a repetition or an observable change in the technical execution of the back-squat increasing injury risk (e.g., spinal flexion, valgus collapse, asymmetry, and imbalance).

### Neuromuscular Function

#### Knee Extensor Strength

An isokinetic dynamometer (Contrex, Dubendorf) was used to determine isometric, concentric, and eccentric strength during a maximal voluntary contraction (MVC). Subjects were seated at a 90° hip angle with the rotational axis of the dynamometer aligned with the lateral femoral epicondyle and a shin pad attached 3 cm proximal to the lateral malleolus. Strapping was applied to the chest, pelvis and mid-thigh to isolate the knee extensor action. Following a 100 w 5 min cycle and three practice contractions, a 5 s isometric MVC was performed 60° below full extension. Isokinetic concentric and eccentric dynamic MVCs were performed from 90° below full extension to full extension at a velocity of 90°⋅s^-1^. At least three valid MVCs were performed for each contraction type, with 3 min rest between attempts. Before each attempt verbal encouragement was given with subjects instructed to push as fast and forcefully as possible. For isometric strength the highest peak torque value was used as the criterion for temporal comparison. The largest area under the torque-time curve [i.e., work (J)] was used as the criterion for the dynamic MVCs. To measure series compliance, the angle of peak torque from the concentric MVC was used as the criterion for comparison (Brockett et al., 2001).

#### Countermovement Jump Height

Countermovement jump (CMJ) height was measured between infrared timing gates (OptoJump, Bolzano). Jump height was calculated from flight time (Komi and Bosco, 1978; Byrne and Eston, 2002). To prevent non-vertical movement between take-off and landing, subjects were instructed to keep arms akimbo and their body straight throughout, landing in the same upright toe-first position as for take-off. Subjects performed three CMJs with 3 min rest between attempts. Peak jump height was used as the criterion measure for temporal comparison.

### Muscle Pain

Subjects were asked to quantify general feelings of lower-body muscle pain immediately following three body-weight squats, marking a 10 cm visual analog scale (VAS). The VAS descriptors were “no pain” (0 cm), to “worst possible pain” (10 cm).

### Serum Creatine Kinase

Venous blood was drawn from the antecubital fossa 0 h, +4, +24, +48 and +72 h following RT. Samples were allowed to clot at room temperature then centrifuged at 3500 rpm for 10 min at 22°C. Serum was then separated and stored at -80°C. Creatine kinase activity (CK) was measured as an indirect marker of muscle damage (Brancaccio et al., 2007). CK was quantified via spectrophotometry using an enzymatically coupled assay (Sigma-Aldrich). From two-level quintuplet controls the coefficient of variation (CV) was calculated as <3% within and <10% between each assay plate. Three basal samples were drawn prior to 0 h to established day-to-day variation, which was shown to be stable over time (CV = 23%) (Ricós et al., 1999).

### Statistics

Data are mean (SD) or [low, high] 90% confidence interval (CI). Data were assessed for normality, sphericity, and homogeneity of variance. Follow-up tests and/or corrections were conducted accordingly. Mixed model analysis of variance (ANOVA) was used to assess time × sex interaction, *post hoc* independent *t*-tests were used to determine sex differences. Repeated measures ANOVA and *post hoc* paired *t*-tests were used to assess change from 0 h for each sex. Effect size estimates (r, r^2^, d, ω^2^, and ω^2^_p_) are reported (Lakens, 2013). Benjamini and Hochberg (1995) false-discovery rate correction was used to correct for familywise error rates. Non-parametric data (muscle pain) was rank transformed for analysis, data are reported as median change from 0 h [interquartile range (IQR)]. From repeated baseline tests the CV was calculated as: 4.3% for CMJ height, 4.4% for isometric strength, 5.1% for eccentric strength, and 2.9% for concentric strength. All statistical tests were performed in RStudio (1.1.383).

## Results

At baseline men were stronger than women [1RM men 118 (14) kg vs. women, 83 (15) kg; *P* < 0.001, *d* = 2.4; Figure 2] lifting greater absolute loads during RT [men, 95 (11) kg vs. women, 66 (12) kg]. When scaled (kg DXA lean mass) no differences were noted between sexes [men 1.9 (0.2) kg⋅kg^-1^ vs. women 1.8 (0.3) kg⋅kg^-1^, *P* = 0.401, and *d* = 0.3; Figure 2]. There was no difference in RT-experience between sexes [men, 2.1 (0.8) years vs. women 2.4 (1.0) years, *P* = 0.746, and *d* = 0.3]. Two female subjects reported taking a combined monophasic oral contraceptive pill with low-dose ethinyl estradiol (30–35 μg) together with progestin (150–200 μg). In the eumenorrheic women menstrual cycle phase was estimated back from the 1st day of bleeding. Baseline (0 h) to +72 h, four women were in the follicular phase (range = days 1–11) and two were in the luteal phase (range = days 15–28) of the menstrual cycle.

No differences were reported for dietary energy intake or protein intake between sexes during the study when scaled to body mass [energy intake 126 (33) kJ⋅kg^-1^⋅d^-1^; protein intake 1.6 (0.3) g⋅kg^-1^⋅d^-1^; pooled mean (SD), *P* > 0.419, and *d* < 0.1].

### Muscle Damage and Pain

Basal CK was lower in women [32 (15) IU⋅L^-1^] than men [80 (42) IU⋅L^-1^, *P* = 0.009, and *d* = 1.3]. Discernable increases in CK were observed +24 h for men [126 (62) IU⋅L^-1^, *P* = 0.054, and *d* = 1.1] and women [89 (41) IU⋅L^-1^, *P* = 0.032, and *d* = 1.3]. There was no sex × time interaction for CK (*P* = 0.313, ω^2^_p_ = 0.04).

Both groups reported increases in muscle pain post-RT at all time-points (*P* < 0.05, *d* > 0.9), +4 h [men, +3 (1) cm; women, +5 (2) cm], +24 h [men, +5 (2) cm; women, +5 (1) cm), +48 h [men, +4 (1) cm; women, +5 (1) cm], and +72 h [men, +2 (2) cm; women, +3 (1) cm]. Women reported greater lower-body muscle pain compared to men at +4 h (*P* = 0.005, *d* = 2.0).

### Knee Extensor Strength

**FIGURE 3:**
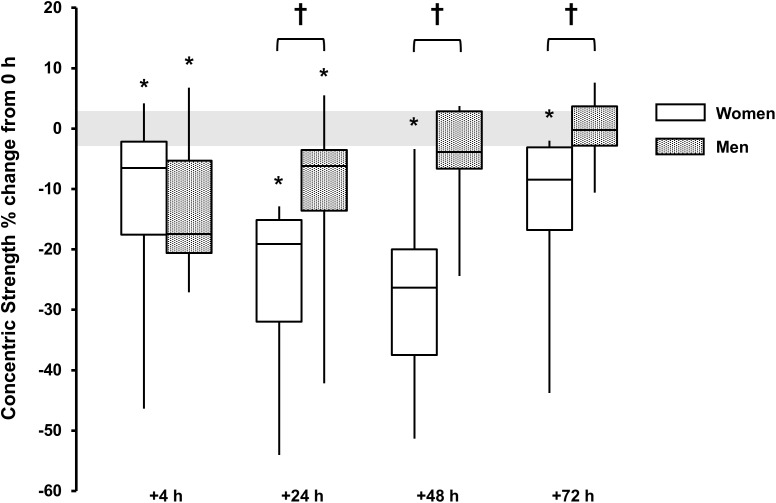
Concentric strength % change from baseline (0 h) between men (gray boxes) and women (open boxes). Boxplot shows median (line) and interquartile range (IQR) (box), whiskers represent the maximum and minimum values. Gray shaded area is the measurement coefficient of variation (CV) (2.9%). The symbol ‘**^†^**’ indicates a difference between sexes; The symbol ‘^∗^’ indicates a difference from 0 h.

For concentric strength a main effect for time was observed for both sexes (*P* < 0.006, ω^2^ > 0.23) with strength loss observed at +4 h [men, -13 (-19, -7)%, *P* = 0.008, and *d* = 1.3; women, -13 (-28, 2)%, *P* = 0.079, and *d* = 0.7], +24 h [men, -10 (-17, -3)%, *P* = 0.010, and *d* = 0.8; women -25 (-35, -16)%, *P* = 0.006, and *d* = 1.8], +48 h and +72 h for women [48 h, -27 (-38, -16)%, *P* = 0.008, and *d* = 1.7; 72 h, -14 (-25, -4)%, *P* = 0.032, and *d* = 0.9]. There was a sex × time interaction (*P* < 0.001, ω^2^_p_ = 0.25) with sex differences at +24 h (*P* = 0.060, *d* = 1.1), +48 h (*P* = 0.003, *d* = 1.9) and +72 h (*P* = 0.042, *d* = 1.3) (Table 1 and Figure 3).

**Table 1:** Change in muscle function in response to resistance training for maximal voluntary contraction (MVC) strength and counter-movement jump (CMJ) height.

	Baseline (0 h)	+4 h	+24 h	+48 h	+72 h
**Concentric MVC (J)**			
Men	167 (32)	145 (31)^∗^	153 (42)^∗^**^†^**	163 (40)**^†^**	168 (34)**^†^**
Women	104 (23)	87 (10)^∗^	75 (9)^∗^**^†^**	74 (13)^∗^**^†^**	86 (14)^∗^**^†^**
**Isometric MVC (N·m)**			
Men	276 (67)	246 (60)^∗^	256 (68)	268 (74)	276 (60)
Women	177 (51)	147 (31)^∗^	139 (24)	142 (22)	151 (25)
**Eccentric MVC (J)**			
Men	202 (39)	168 (27)^∗^	160 (41)^∗^	175 (37)^∗^	188 (43)
Women	119 (32)	100 (22)^∗^	89 (18)^∗^	95 (25)^∗^	110 (26)
**CMJ Height (cm)**			
Men	38 (5)	33 (5)^∗^**^†^**	34 (4)^∗^**^†^**	35 (6)^∗^**^†^**	37 (6)**^†^**
Women	25 (2)	20 (2)^∗^**^†^**	20 (3)^∗^**^†^**	20 (3)^∗^**^†^**	22 (4)^∗^**^†^**

***^†^**indicates a difference between sexes; ^∗^indicates a difference from 0 h.*

Angle of peak torque 0 h was 74 (6)° below full extension for men and 63 (6)° for women. There was no main effect for time for either sex (*P* > 0.442, ω^2^ < 0.02) or sex × time interaction (*P* = 0.538, ω^2^_p_ = 0.02).

**FIGURE 4:**
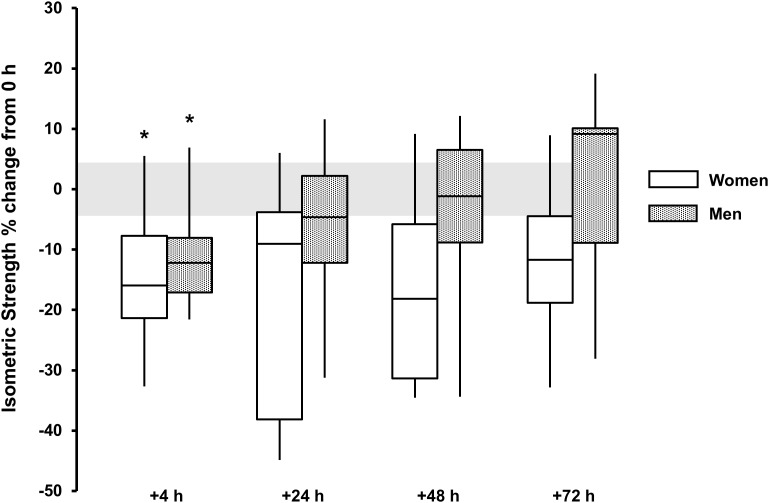
Isometric strength % change from baseline (0 h) between men (gray boxes) and women (open boxes). Boxplot shows median (line) and IQR (box), whiskers represent the maximum and minimum values. Gray shaded area is the measurement CV (4.4%). The symbol ‘^∗^’ indicates a difference from 0 h.

For isometric peak torque a main effect for time was observed for both sexes (*P* < 0.015, ω^2^ > 0.19). Torque loss was observed only at +4 h for men [-10 (-17, -3)%, *P* = 0.009, and *d* = 1.2] and women [-15 (-25, -5)%, *P* = 0.054, and *d* = 1.2]. There was no discernable sex × time interaction (*P* = 0.135) (Table 1 and Figure 4).

**FIGURE 5:**
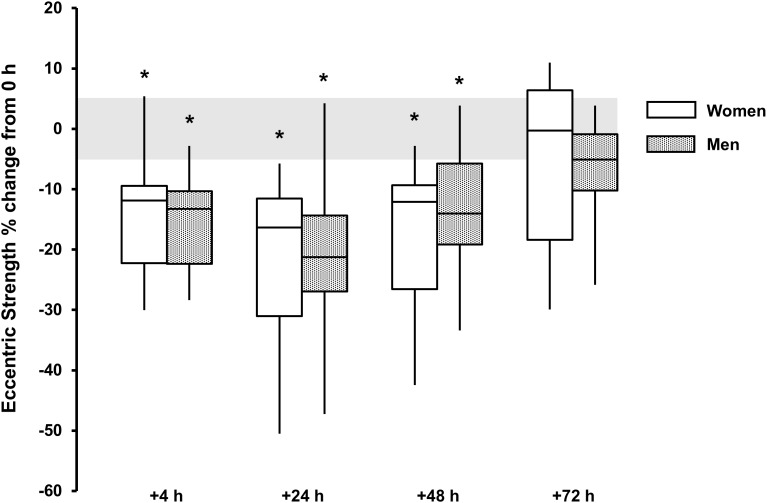
Eccentric strength % change from baseline (0 h) between men (gray boxes) and women (open boxes). Boxplot shows median (line) and IQR (box), whiskers represent the maximum and minimum values. Gray shaded area is the measurement CV (5.1%). The symbol ‘^∗^’ indicates a difference from 0 h.

For eccentric strength a main effect for time was observed for both sexes (*P* < 0.002, ω^2^ > 0.33) with strength loss observed at +4 h [men, -16 (-20, -12)%, *P* < 0.001, and *d* = 2.0; women, -14 (-21, -6)%, *P* = 0.024, and *d* = 1.3), +24 h [men, -21 (-28, -14)%, *P* < 0.001, and *d* = 1.7; women, -21 (-32, -12) %, *P* = 0.017, and *d* = 1.5] and +48 h [men, -13 (-19, -7)%, *P* = 0.006, and *d* = 1.2; women, -18 (-29, -8)%, *P* = 0.025, and *d* = 1.2]. There was no sex × time interaction (*P* = 0.497, ω^2^_p_ = 0.04) (Table 1 and Figure 5).

### Countermovement Jump Height

**FIGURE 6:**
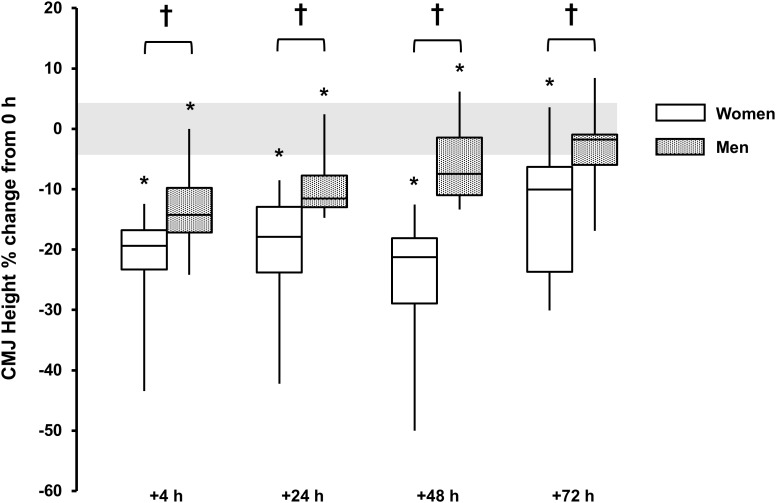
Countermovement jump height % change from baseline (0 h) between men (gray boxes) and women (open boxes). Boxplot shows median (line) and IQR (box), whiskers represent the maximum and minimum values. Gray shaded area is the CV for the measurement error (4.3%). The symbol ‘**^†^**’ indicates a difference between sexes; The symbol ‘^∗^’ indicates a difference from 0 h.

A main effect for time reported for both sexes (*P* < 0.001, ω^2^ > 0.49), with decrements observed +4 h [men, -14 (-17, -10)%, *P* < 0.001, and *d* = 1.9; women, -22 (-28, -15)%, *P* = 0.002, and *d* = 2.2], +24 h [men, -10 (-13, -7)%, *P* < 0.001, and *d* = 1.7; women, -20 (-27, -13)%, *P* < 0.001, and *d* = 1.8] and +48 h [men, -6 (-10, -3)%, *P* = 0.016, and *d* = 1.0; women, -25 (-33, -17) %, *P* = 0.001, and *d* = 2.0] and +72 h for women [-13 (-21, -5)%, *P* = 0.015, and *d* = 1.1]. There was a sex × time interaction (*P* < 0.001, ω^2^_p_ = 0.21) with sex differences at +4 h (*P* = 0.048, *d* = 1.0), +24 h (*P* = 0.037, *d* = 1.1), +48 h (*P* < 0.001, *d* = 2.1), and +72 h (*P* = 0.051, *d* = 1.1) (Table 1 and Figure 6).

## Discussion

The main finding from this study was that following lower-body RT, performed to a predefined level of volitional exhaustion, women had a more pronounced loss and prolonged recovery of neuromuscular function compared to their male counterparts. Different temporal recovery patterns were observed between functional measures for both sexes. For example, the loss of isometric strength was resolved 1–2 days earlier than the dynamic measures of strength, and we did not observe any sex differences for eccentric MVC strength, although a loss of strength was still observed up to +72 h. We suspect that the differences in the temporal recovery between functional measures may be related to the mode of RT with the CMJ and concentric strength measures more closely related to the RT-exercise. Indeed, the back-squat exercise is a dynamic multiarticular movement requiring activation and co-ordination of several muscle groups (Schoenfeld, 2010). The back-squat load and total volume during RT was determined from the 1RM (i.e., inability to lift 80% 1RM). At baseline knee extensor concentric MVC strength was the strongest predictor of 1RM back-squat strength (*r*^2^ = 0.540, *P* < 0.001) above all other measure of function (*P* > 0.206). Thus, it seems plausible that concentric MVC strength would also be a more sensitive and specific measure of fatigue in response to a RT-session using the back-squat exercise. The CMJ was also a strong predictor of 1RM strength at baseline (*r* = 0.7, *P* = 0.001), however, only moderate correlation was observed between the loss CMJ height and the loss concentric MVC strength (*r* = 0.4, *P* = 0.098), yet both measures followed similar temporal recovery patterns. We posit that outside of the knee extensor fatigue the CMJ may have additionally captured the fatigue-response of the other muscle groups (e.g., hip extensors, erector spinae, and abdominals) recruited during the back-squat exercise.

The absence of a robust CK response [+24 h, 74 (41) IU⋅L^-1^; pooled mean (SD)] and change in series compliance indicate low-level of disruption to the muscle in response to RT (Brockett et al., 2001; Brancaccio et al., 2007), which is likely due to subject’s RT-experience and familiarity with the exercise (Newton et al., 2008; Hyldahl et al., 2017). However, decrements were still noted +4 to +72 h for all functional measures. Indeed, a robust pain response was reported and the small perturbation in CK +24 h indicates some minor disruption to the muscle milieu (MacIntyre et al., 2000; Crameri et al., 2007; Allen et al., 2008; Place et al., 2015), however, these responses cannot explain the difference in temporal recovery between men and women.

During exercise sex differences for neuromuscular fatigue are principally thought to reside in the muscle (Hunter et al., 2004, 2006; Wüst et al., 2008; Ansdell et al., 2017). In a comparable study, it was reported that women had a greater loss of MVC torque +24 and +48 h following eccentric dorsiflexor exercise, compared to men (Power et al., 2013). It was shown that the women also had a more pronounced loss of force at a low-stimulation frequencies, indicating a greater disruption of the slow-muscle type, which assumedly constitutes a higher proportion in the women (Miller et al., 1993; Staron et al., 2000). In the absence of any change to series compliance, or change in voluntary activation, the authors speculated that greater excitation-contraction uncoupling and impaired Ca^2+^ release occurred in women causing prolonged recovery of strength compared to the men (Power et al., 2013).

It is also possible that sex differences in this study may be due to different central responses between men and women. Central fatigue following damaging-exercise, has been shown to affect muscle force +2 to +168 h (Prasartwuth et al., 2005; Goodall et al., 2016). The early onset of fatigue in men *vs.* women during exercise has been associated with larger reductions in voluntary activation (Martin and Rattey, 2007), which is likely due to the higher intramuscular pressure and metabolite accrual in the stronger male muscle. Therefore, the fatigue-related afferent feedback during RT could have been greater for the men than the women (Russ and Kent-Braun, 2003). This would ostensibly lead to an earlier exercise cessation in the men, enabling a more rapid long-term recovery. There is limited research investigating effect of sex on central fatigue, which has only been examined during exercise and a few minutes following cessation (Hunter et al., 2006; Martin and Rattey, 2007). To our knowledge, the full temporal pattern of recovery hours or days following exercise has not been investigated. Based on the results reported in this paper, and the substantiated sex differences in pain response and pain perception to exercise (Racine et al., 2012), further research partitioning central and peripheral aspects of fatigue is apt.

Early research has showed that female strength levels vary with menstrual cycle phase, peaking mid-cycle when estrogen levels are high (Sarwar et al., 1996). However, further research has not supported these early findings showing that strength and fatigability are not influenced by menstrual cycle phase or use of oral contraceptives (Hunter, 2014). Menstrual cycle phase was not controlled in this study with 50% of the women in the follicular phase, 25% in luteal phase and 25% taking oral contraceptive pills during the RT-day and next 3 days of recovery. To our knowledge there is no research investigating the effect of the menstrual cycle phase on the long-term recovery from exercise. Enhanced recovery has been reported in the follicular (high estrogen) phase of the menstrual cycle compared to women in the luteal phase (low estrogen), +96 and +168 h following exercise-induced muscle damage (Markofski and Braun, 2014). Indeed, a potential prophylactic effect has been reported for estrogen, blunting muscle damage and preserving muscle function following exercise-induced muscle damage (Minahan et al., 2015). We observed no robust change in CK (a marker of muscle damage), and thus, we are not aware how the phase of menstrual cycle could have affected the temporal recovery of muscle function in absence of any overt muscle damage. And although we are limited by our experimental design, sample size and the *post hoc* group allocation, we have no evidence to suggest the menstrual cycle phase affected the temporal recovery of function in this study.

Prior RT-experience has been shown to improve the temporal recovery of neuromuscular function following RT (Newton et al., 2008; Hyldahl et al., 2017). For this study, we recruited subjects with similar RT-experience [men, 2.1 (0.8) years vs. women 2.4 (1.0) years]. As a result, men and women had similar relative levels of strength [i.e., per kg lean mass, men 1.9 (0.2) kg⋅kg^-1^ vs. women 1.8 (0.3) kg⋅kg^-1^], but different absolute levels of strength [men 118 (14) kg vs. women, 83 (15) kg]. If we were to match men and women for absolute strength, on average, the women would have more RT-experience, which may also affect temporal recovery from RT (Newton et al., 2008; Hyldahl et al., 2017). Therefore, we do not know to what extent the sex difference was caused by the difference in absolute strength, and we are not sufficiently powered to conduct *post hoc* analysis, accounting for this (Ansdell et al., 2017). For future research matched-pair design blocking training-experience and strength would be a more powerful approach. Additionally, further measures are necessary to provide direct insight into the cause of sex difference (i.e., fiber typing and partitioning central and peripheral aspects of fatigue).

Knowledge pertaining to the temporal recovery of neuromuscular function allows practitioners to make more informed decisions when designing longitudinal training programs. Based on the findings from this study, we suggest the sex of the athlete should be considered when planning RT during periods of intense training or in temporal proximity to performance. To conclude, sex differences were observed in the temporal recovery of neuromuscular function +4 to +72 h following RT. These differences are explained by differences in RT load, intensity, fatigability, experience, strength, or muscle damage, but may also be related to differences in muscle phenotype and/or central mechanisms.

## Author Contributions

RD, BC, and PJ designed the research project and had primary responsibility for the final content of the manuscript. RD conducted the research, analyzed the data, and drafted the manuscript. All authors read and approved the final manuscript.

## Conflict of Interest Statement

The authors declare that the research was conducted in the absence of any commercial or financial relationships that could be construed as a potential conflict of interest.
